# Supplementary material for: The causal relationship between the human gut microbiota and pyogenic arthritis: a Mendelian randomization study
Source: Front Cell Infect Microbiol. 2024 Nov 26;14:1452480. doi: 10.3389/fcimb.2024.1452480 (PMC11629706; doi:10.3389/fcimb.2024.1452480)
Supplement: Supplementary file 1 [file DataSheet1.zip › mendelian test/ebi-a-GCST90027669.csv_leave_one_out.pdf]

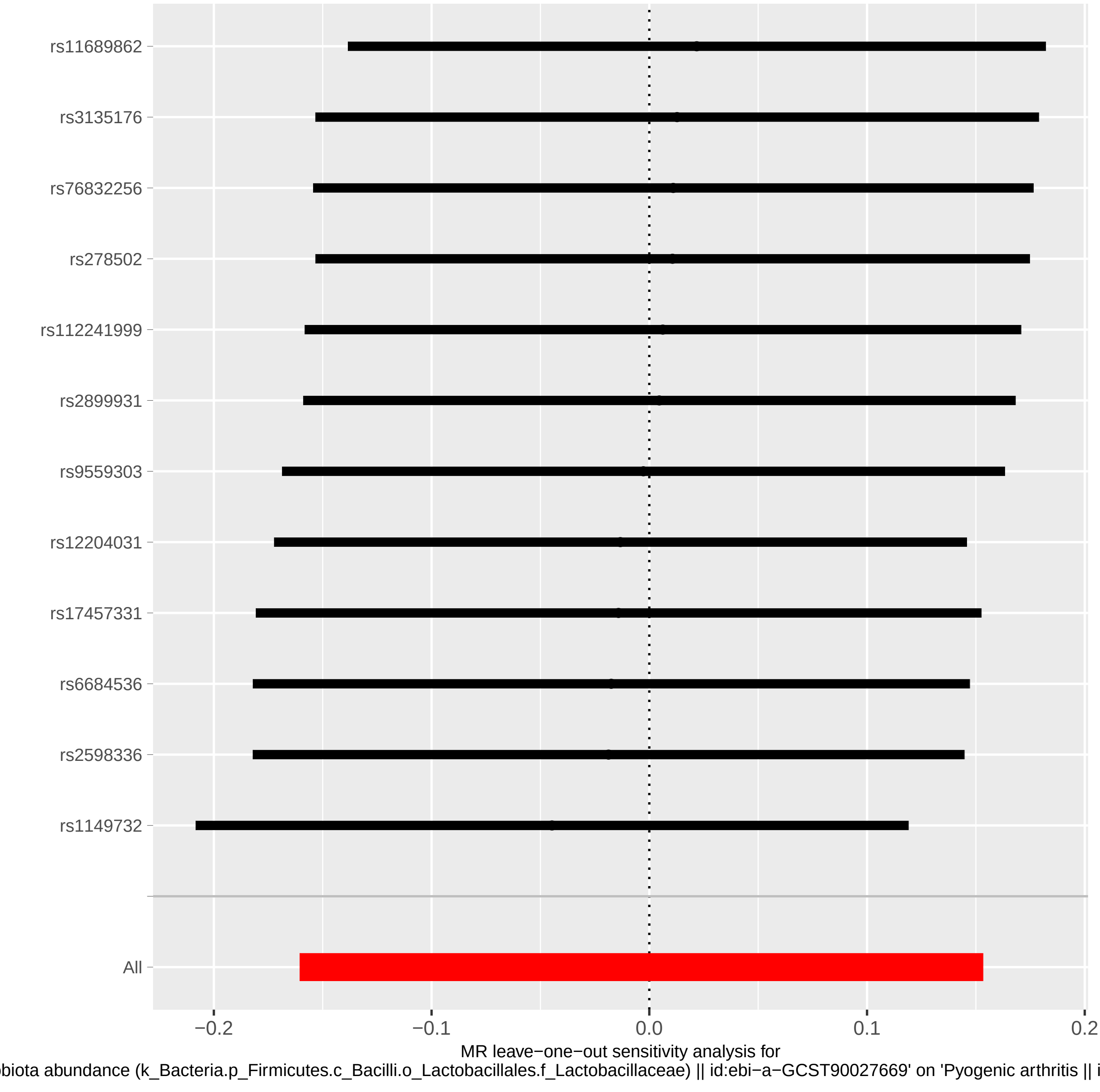

rs11689862
rs3135176
rs76832256
rs278502
rs112241999
rs2899931
rs9559303
rs12204031
rs17457331
rs6684536
rs2598336
rs1149732
All
−0.2
−0.1
0.0
0.1
0.2
MR leave−one−out sensitivity analysis for
biota abundance (k_Bacteria.p_Firmicutes.c_Bacilli.o_Lactobacillales.f_Lactobacillaceae) || id:ebi−a−GCST90027669' on 'Pyogenic arthritis || i
